# Western and Modern Mexican dietary patterns are directly associated with incident hypertension in Mexican women: a prospective follow-up study

**DOI:** 10.1186/s12937-018-0332-3

**Published:** 2018-02-14

**Authors:** Adriana Monge, Martín Lajous, Eduardo Ortiz-Panozo, Beatriz L. Rodríguez, José Juan Góngora, Ruy López-Ridaura

**Affiliations:** 1 Center for Research on Population Health, National Institute of Public Health, Cuernavaca, Mexico; 2 Escuela de Medicina, Tecnológico de Monterrey, Monterrey, Mexico; 3 University of Hawaii at Manoa, Honolulu, HI USA; 4 Department of Global Health and Population, Harvard T.H. Chan School of Public Health, Boston, USA

**Keywords:** Diet, Dietary patterns, Hypertension, Women, Mexican

## Abstract

**Background:**

Research has found that diet and dietary patterns are associated with blood pressure and hypertension. Limited research in this area has been conducted in a Mexican population.

**Methods:**

We investigated the relation between dietary patterns (principal component analysis) and the incidence of self-reported treated hypertension in 62,913 women from the Mexican Teachers’ Cohort, a large population-based cohort of female Mexican teachers, who were free of hypertension at baseline in 2006–2008 when diet was assessed using a food frequency questionnaire. Dietary patterns were categorized into quartiles and logistic regression models were fit.

**Results:**

Participants were 42.1 ± 7.3 years old, had a BMI 27.0 ± 4.4 and a cumulative incidence of hypertension of 4.6%. Between baseline and first follow-up (2011–2014) we identified 2916 incident cases of hypertension. We identified three major components. The first was loaded heavily with vegetables, fruits and legumes; the second component was loaded heavily with processed meats, fast foods, and red meat; and finally the third component was loaded heavily with corn tortillas, hot peppers, and sodas. We named the components as Fruits & Vegetables (FV), Western (W), and Modern Mexican (MM).

The multivariable-adjusted odds of hypertension in the highest quartile of the W pattern were 24% higher than the odds for individuals in the lowest quartile (95%CI = 1.10, 1.40; *P*-trend = 0.0004); women in the highest quartile of the MM pattern had 15% higher odds than women in the lowest quartile (95%CI = 1.02, 1.29; *P*-trend = 0.01). The FV pattern was not significantly associated with hypertension (OR for extreme quartiles = 0.94; 95%CI = 0.84, 1.05; *P-*trend = 0.19).

**Conclusion:**

The Western pattern and the Modern Mexican pattern, which showcases an undergoing nutrition transition, may affect the incidence of hypertension, whereas the FV pattern was not associated with hypertension. These findings are important in the prevention of hypertension and cardiovascular diseases in Mexico and possibly among Mexican people living in the US.

**Electronic supplementary material:**

The online version of this article (10.1186/s12937-018-0332-3) contains supplementary material, which is available to authorized users.

## Background

Hypertension is an important public health concern in Mexico where the prevalence in adult women is 30% [1]. High blood pressure is a marker of cardiovascular risk and has important health impacts, such as on cardiovascular and cerebrovascular disease, renal failure, retinopathy and optic neuropathy. Diet is a modifiable risk factor for hypertension, however results from studies on individual foods and nutrients (i.e. coffee and caffeine, dairy, cruciferous vegetables, etc.) have been controversial [2–8]. Evaluating dietary patterns has been proposed as a strategy that captures the overall manner in which individuals consume foods [9–11]. Dietary patterns overcome the challenge of collinearity of studying single foods or nutrients and potentially considering their joint effects [10].

Dietary patterns based on a priori indices have been associated to decreased risk of chronic disease. While there is robust scientific evidence on the benefits of the DASH diet (Dietary Approaches to Stop Hypertension) and the Mediterranean diet on lowering blood pressure [12–14], these a priori indices have been generated from studies done mostly in Caucasian populations, with little representation of Mexicans, one of the largest minorities in the US. It is questionable whether these indices are appropriate for the Hispanic population, specifically Mexicans, since evidence shows low adherence to these indices [15]. In contrast, empirically derived dietary patterns are data driven and allow for the examination of eating behaviors without prior knowledge or assumption of the existence of dietary patterns in the population.

There is a need to understand dietary patterns within the Mexican population and comprehend which of these patterns are associated with hypertension risk and subsequently target culturally appropriate public health messages of healthy diets. In this study we derived dietary patterns using principal component analysis to explore the association between the Mexican dietary patterns and incident hypertension in a population-based study.

## Methods

### Study population

The Mexican Teachers’ Cohort (MTC) was established in 2006–08, when 115,315 female teachers aged 25 years and older responded to a scannable paper questionnaire on demographic and reproductive characteristics, diet, lifestyle, and medical conditions. Women participate in a well-established voluntary economic incentives program and the study questionnaire was delivered and collected in collaboration with public education authorities from 12 states in Mexico [16]. All women signed an informed consent form to take part in the study. This study was approved by the Institutional Review Board at National Institute of Public Health of Mexico (INSP). Between December 2011 and February 2014, a follow-up questionnaire was released. Sixty-nine percent of participants responded to a paper-based questionnaire, 2% to an online questionnaire and 11% to a short telephone interview for an overall response of 82% for the 2011–2014 questionnaire.

After a feasibility phase in 2006, 106,466 participants responded a questionnaire in 2008 that included a previously validated dietary questionnaire [17]. We excluded women with unrealistic energy intake (500 > calories> 3500) or an invalid questionnaire (70 or more items missing, or cereals and grains section missing) (*n* = 18,396). Prevalent cases of hypertension (*n* = 11,292), myocardial infarction, stroke or cancer (*n* = 1772) were also excluded. Finally, we excluded 12,093 participants for whom information after baseline was unavailable. The final study sample included 62,913 participants.

### Dietary assessment

We collected dietary information with a 140-item semi-quantitative food frequency questionnaire (FFQ). Participants were asked to specify the average frequency of consumption over the previous year of each food item in a commonly used unit or portion size. The consumption frequencies were never, once a month or less, two to three times a month, once a week, two to four times a week, five to six times a week, once a day, two to three times a day, four to five times a day and six or more times a day. With the USDA food-composition database [18] and the database used in the National Health and Nutrition Survey in Mexico we calculated nutrient and energy intakes by multiplying the nutrient content of the pre-defined portion sizes by the frequency of consumption. A similar version of our FFQ was previously validated amongst 134 Mexico City female residents in a 12-month study [17]. Pearson correlation coefficients for total energy, carbohydrate, protein, and total fat intakes between the FFQ and four 4-day 24-h recalls were 0.52, 0.57, 0.32, and 0.63, respectively.

### Hypertension assessment

At follow-up, we asked participants to report whether a clinician had made a diagnosis of elevated blood pressure in the previous two years, if treatment was received, and the year of diagnosis. We defined hypertension as self-reported physician diagnosed elevated blood pressure under drug treatment. We assessed the validity of self-reported hypertension diagnosis in a random subsample of 101 participants who reported elevated blood pressure, using a structured phone interview to confirm the diagnosis, year of diagnosis and treatment. We confirmed the presence of hypertension in 89% of participants who reported a clinical diagnosis of elevated blood pressure under treatment, whereas only 50% of cases were confirmed in those who reported a diagnosis without treatment.

### Dietary patterns

Dietary patterns were derived using all valid FFQs at baseline (*n* = 88,082). Food items in the FFQ were collapsed into 37 food groups (Table 1) based on similarity of nutrient content by a trained dietitian. Some individual foods were retained because they represented distinct dietary patterns or constituted a distinct item on their own (e.g. corn tortillas, atole, and eggs). Gram amounts of food groups were converted to calories and divided by total energy intake of each individual, resulting in food groups standardized as percent of total energy intake plus one and log transformed to normalize the distribution.

**Table 1:** Thirty-seven food groups derived from the 140-item food frequency questionnaire of the Mexican Teachers’ Cohort^a^

Food group	Description
Carrots	Carrots
Tomato	Red tomato cooked and raw
Green vegetables	Lettuce, dark leafy greens, broccoli or cauliflower, cabbage
Starchy vegetables	Potatoes and corn
Other vegetables	Onions, tomatillo, cucumber, squash/chayote, avocado, nopal, jicama, green beans, beets, pumpkin flowers
Hot peppers	Hot peppers sauce, hot peppers canned, dried hot peppers
Fruits	Lime, orange/mandarin, apple, papaya, banana, mango, guava, pear, pineapple, melon, watermelon, apricot, grapes, prickly pear, strawberries, grapefruit, raisins, plum, mamey, sapodilla
Legumes	Beans, Green peas, lentils, broad beans
Nuts	Peanuts, walnuts, almonds - unprocessed
Milk and yogurt	Whole, skim and semi milk, soy milk, yogurt or bulgurs
Cheese	Fresh cheese, Oaxaca, Cream cheese, Other types of cheese, Manchego/Chihuahua cheese
Egg	Egg
Seafood	Canned tuna, white fish, shrimp, fatty fish, other seafood, canned sardines, and dry fish
Poultry	Chicken
Red meat	Beef, pork, barbacoa, carnitas, dry beef, birria
Organs	Liver, pancita or menudo
Pozole	Pozole
Corn tortilla	Corn tortilla
Pasta and rice	Rice and pasta
Refined grains	French style baguette, ready to eat cereals, white loaf of bread, wheat flour tortilla, saltine crackers
Whole grain	Whole loaf of bread, high fiber ready to eat cereals
Atole	Atole with and without milk
Breakfast and cereal bars	Oats, granola bars
Candies and jams	Candy, jams, chocolate
Pastries	Sweet bread, cookies, donuts, cakes
Milk desserts	Jello or flan, fermented milk drink, Ice cream, petit suisse,
Fast foods	Pizza, hotdog, hamburguers
Meat by products	Turkey ham, pork ham, sausage, chorizo/longaniza, pork skins, bacon, other cold meats
Antojitos	Sopes/quesadillas, tacos, tamales, tortas
Butter, margarine and cream	Cream, butter, margarine
Snacks	Fried snacks, processed snack nuts
Coffee and tea	Regular or decaf coffee and tea
Soda	Regular soda
SSBs	Fruit waters, hibiscus water
Juice	Orange juice
Alcohol	Beer, tequila, brandy, whisky, rum, pulque, mezcal, aguardiente
Wine	Wine

^a^Foods within food groups appear in increasing frequency of consumption

We defined dietary patterns on the 37 food groups with principal component analysis (PCA) using the factor procedure in SAS. PCA reduces the number of observed variables to a smaller number of principal components that account for most of the variance of the observed variables. Components were rotated by an orthogonal transformation (*Varimax* rotation function) to achieve simpler structures with greater interpretation. The number of components retained were determined using a combination of the eigenvalues (> 1.5), the scree plot, and the interpretability of the components. The cut-off value for eigenvalues is arbitrary, some have used eigenvalues > 1.25 [11, 19] however, the usual value is > 1.0 but this cut-off generated too many factors. We decided not use the percentage of variance explained to determine the number of components to retain because this depends on the number of variables entered into the analysis [20, 21]. There are two approaches to calculate the factor scores per participant, one is to compute the score by summing the observed intakes of the component food items weighted by its factor loading. The second approach is a simpler approach to sum only the factors that load higher than a certain value [19]. We chose to use the more elaborate approach to calculate factor scores for this analysis (results were similar). Factor loadings >|0.30| were considered to contribute to the component and were used to name the dietary patterns.

### Covariates

Covariate information was based on self-reports from the baseline questionnaire. Responses to ownership of seven household assets (phone, car, computer, vacuum cleaner, microwave oven, cell phone and internet) were summed and classified into low, medium and high socioeconomic status tertile. Participant’s education was classified as having a high-school degree or less, an undergraduate degree, or a graduate degree or higher. To account for income and health disparities between different regions in Mexico we categorized individuals based on their State of residence (Baja California, Chiapas, Durango, Guanajuato, Hidalgo, Jalisco, Mexico City, Mexico State, Nuevo Leon, Sonora, Yucatan and Veracruz). Physical activity was defined as self-reported minutes per week spent on moderate and vigorous recreational physical activity calculated from responses to eight categories that ranged from none to more than 10 h per week. Menopausal status was based on self-reported information related to last menstruation, hot flushes, hysterectomy, oophorectomy, and hormonal treatments; if these data were unknown then an algorithm using current age was used to determine status where possible. Smoking status was classified into never, past or current smokers. Diabetes and hypercholesterolemia were defined by self-reported treated disease. Body mass index (BMI) was calculated as self-reported weight in kg over height in meters squared.

### Statistical analyses

Continuous variables were summarized as means ±SD and categorical variables as percentages. Our main exposures were the three major dietary patterns derived from PCA. We categorized individuals into quartiles of each dietary pattern using the lowest category as the referent. We estimated age-adjusted and multivariable-adjusted odds ratios (OR) and 95% confidence intervals (CI) using logistic regression models (SAS 9.4, SAS Institute Inc., Cary, NC). The median value for each quartile was used as a continuous variable to test for linear trend. A complete-case analysis was conducted.

In multivariable models we adjusted for age (continuous), socioeconomic status (tertiles), education (high-school or less, undergraduate, graduate or more), state of residence (12 States), menopausal status (premenopausal, postmenopausal, unknown), smoking (never, past, current, and unknown), recreational physical activity (minutes/week), diabetes, hypercholesterolemia, and energy intake (quartiles). In an exploratory model we also adjusted for BMI (kg/m^2^) as a potential mediator to conduct an exploratory mediation analysis.

In sensitivity analyses we conducted the analyses among non-diabetics because diabetics could have altered their diet after diagnosis but they are still at a higher risk of hypertension. We also repeated analyses among never-smokers because smoking is a strong risk factor for hypertension and could mask the association between diet and hypertension. To take into account that deriving dietary patterns through PCA has arbitrary decisions which may affect the reproducibility of the findings, we repeated the analyses 1) rotating the patterns using an oblique factor rotation (*promax* rotation), 2) retaining different number of factors (*n* = 2), 3) using all individual food items (*n* = 140) instead of collapsing into food groups, 4) running the analysis using the maximum likelihood method as an alternate method to PCA, and 5) dividing the whole cohort randomly into two groups to show whether the major dietary patterns were similar for the two groups and closely resembled those for the overall sample. The two component solution was used as an alternative exposure in sensitivity analyses. To assess whether our findings were sensible to the hypertension definition, we also used an alternative definition of hypertension as those participants who responded having had a diagnosis of hypertension regardless of medication use (*n* = 3650 incident cases).

## Results

**Fig. 1:**
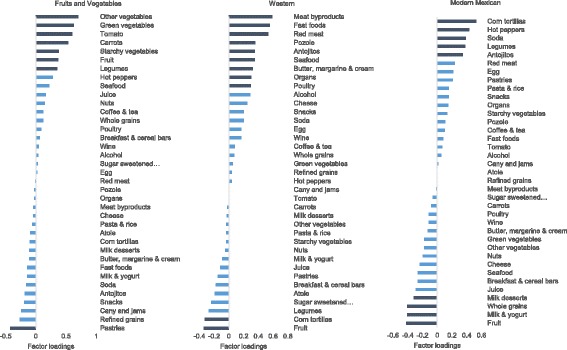
Factor loadings of dietary patterns derived from the Mexican Teachers’ Cohort. Rotated factor loadings of the three dietary patterns derived in 88,082 Mexican women who responded the 2008 food frequency questionnaire from the Mexican Teachers’ Cohort. The first dietary pattern was named Fruits and Vegetables, the second Western, and the third Modern Mexican. The variability explained by each factor was 8.6%, 5.9% and 5.1%

The mean age of participants was 42.1 ± 7.3 years. We identified 2916 incident cases of hypertension and three dietary patterns. The cumulative incidence of hypertension was 4.6% over 1.7 ± 1.1 years. Factor-loading matrixes for these patterns are shown in Fig. 1, positive loadings indicate a positive association with the component, while negative loadings indicate an inverse association with it. The first component was loaded positively with other vegetables, green vegetables, tomatoes, carrots and fruits, while pastries loaded negatively; the second component was loaded positively with processed meats, fast foods, and red meat, and negatively with fruit and corn tortilla; and finally the third component was loaded positively with corn tortillas, hot peppers and sodas, and negatively on whole grains, dairy and fruits. The first component explained 8.6% of the total variance, while the second and third component explained 5.9% and 5.1% of the total variance, respectively. We named the component as Fruits & Vegetables, Western, and Modern Mexican. PCA performed on the FFQs responded during the feasibility phase in 2006 resulted in similar dietary patterns.

Age-adjusted baseline characteristics of participants according to quartiles of each dietary pattern are shown in Table 2. Women in the highest quartile of the Fruits & Vegetables pattern were more likely to come from Northern Mexico and less from Mexico City, were more likely to have a graduate education, diabetes and hypercholesterolemia, and reported a higher physical activity than women in the lowest quartile. In the Western pattern, women with the highest category were less likely to be indigenous, more likely to be from Northern Mexico and less from the south and have a higher socioeconomic status and graduate education, they were also more likely to be current smokers and have a lower energy intake. In contrast, women in the highest quartile of the Modern Mexican pattern were more likely to be indigenous, live in Northern and Southern Mexico, and be current smokers, however they were less likely to live in Mexico City, they had a lower socioeconomic status, and reported lower physical activity and energy intake.

**Table 2:** Age-adjusted characteristics of 62,913 Mexican women from the MTC at baseline by dietary pattern quartiles^a-d^

	Fruits & Vegetables Dietary Pattern		Western Dietary Pattern		Modern Mexican Dietary Pattern	
	Q1	Q4	Q1	Q4	Q1	Q4
Age at questionnaire^b^, years	41.1 ± 7.3	43.2 ± 7.1	42.7 ± 7.3	41.3 ± 7.4	42.9 ± 7.5	41.5 ± 7.1
Indigenous^c^, %	7	7	15	5	5	11
Regions in Mexico						
- Northern Mexico, %	17	23	12	31	15	26
- Central Mexico, %	16	18	17	13	18	14
- Mexico City and State of Mexico, %	30	22	21	25	33	17
- Southern Mexico, %	37	37	50	31	34	42
Graduate education, %	13	17	11	17	15	13
Highest socioeconomic status tertile, %	41	47	28	56	47	40
Menopausal status						
- Premenopausal, %	77	76	75	78	76	77
- Postmenopausal, %	14	14	15	13	14	14
- Missing, %	9	10	10	9	10	9
Body mass index, kg/m^2^	27.3 ± 4.6	26.9 ± 4.2	26.7 ± 4.2	27.2 ± 4.6	26.3 ± 4.0	27.7 ± 4.7
Current smokers, %	10	9	4	15	7	12
Diabetes mellitus, %	2	4	3	3	2	4
Hypercholesterolemia, %	9	12	11	10	11	10
Recreational physical activity, min/week	30(0,180)	90(0,270)	60(0,210)	60(0,210)	120(30,330)	30(0,150)
Total energy, kcal/day	1796(1405,2276)	1712(1335,2180)	1825(1409,2322)	1556(1210,1978)	1928(1510,2430)	1550(1216,1961)

^a^Values are mean ± SD for continuous variables and percent for categorical variables. Physical activity and total energy intake are expressed as median (IQR). Values are age standardized to the age distribution of the study population. Values of polytomous variables may not sum to 100% due to rounding. *N* = 15,728 per quartile in all patterns (except Q3: 15,729)

^b^Variable is not age adjusted

^c^Participants who reported speaking an indigenous language or having a parent who did were defined as indigenous

^d^Abbreviations: MTC: Mexican Teachers’ Cohort; Q1-Q4: quartile1-quartile4

Age-adjusted results showed a statistically significant direct association between the Western dietary pattern and the odds of hypertension (Table 3). After adjustment for confounders the association between the Western pattern and the odds of hypertension remained significant. Women in the highest quartile of Western dietary pattern had 24% higher odds of hypertension (95% CI: 1.10, 1.40; *P-*trend 0.0004) relative to those in the lowest quartile. We also adjusted for BMI, a potential mediator of the association between the Western pattern and hypertension. As expected, the association was attenuated but remained significant (OR: 1.17, 95% CI: 1.03, 1.32; *P-*trend 0.03). Adherence to the Modern Mexican pattern was directly associated with the odds of hypertension. Women in the highest quartile of the Modern Mexican pattern had 15% higher odds (95% CI: 1.02, 1.29; *P-*trend 0.01) of hypertension compared to the lowest quartile. After adjusting for BMI the association became null. The Fruits & Vegetables pattern was not significantly associated with hypertension. However, there was an indication of a protective effect when the pattern score was modeled as a continuous variable (OR: 0.96; 95% CI: 0.92, 1.00).

**Table 3:** Multivariate adjusted OR of incident hypertension by dietary pattern quartiles in women from the MTC^a-e^

Model	Q1	Q2	Q3	Q4	*P-*trend	Continuous^a^
Fruits & Vegetables						
Cases	713	737	736	730		
Non-cases	15,015	14,991	14,993	14,998		
Age-adjusted ^b^	1	1.00(0.90,1.11)	0.96(0.87,1.07)	0.91(0.82,1.01)	0.06	0.96(0.92,0.99)
Multivariable ^c^	1	1.03(0.92,1.14)	0.97(0.87,1.09)	0.94(0.84,1.05)	0.19	0.96(0.92,1.00)
Multivariable+BMI^d^	1	1.07(0.96,1.20)	0.99(0.88,1.11)	1.00(0.89,1.13)	0.71	0.98(0.94,1.02)
Western						
Cases	679	729	736	772		
Non-cases	15,049	14,999	14,993	14,956		
Age-adjusted ^b^	1	1.10(0.99,1.22)	1.14(1.02,1.26)	1.23(1.11,1.37)	˂0.0001	1.08(1.04,1.12)
Multivariable ^c^	1	1.10(0.98,1.23)	1.12(1.00,1.26)	1.24(1.10,1.40)	0.0004	1.09(1.04,1.13)
Multivariable+BMI^d^	1	1.10(0.98,1.23)	1.06(0.94,1.20)	1.17(1.03,1.32)	0.03	1.06(1.01,1.11)
Modern Mexican						
Cases	705	752	750	709		
Non-cases	15,023	14,976	14,979	15,019		
Age-adjusted ^b^	1	1.12(1.01,1.24)	1.15(1.03,1.27)	1.10(0.99,1.22)	0.08	1.03(0.99,1.07)
Multivariable ^c^	1	1.11(1.00,1.24)	1.18(1.06,1.32)	1.15(1.02,1.29)	0.01	1.05(1.00,1.09)
Multivariable+BMI^d^	1	1.03(0.92,1.15)	1.08(0.97,1.22)	0.99(0.88,1.12)	0.95	0.99(0.95,1.03)

^a^Multivariate adjusted odds (95% CI) of incident hypertension by dietary patterns (continuous)

^b^Model 1: adjusted for age (continuous)

^c^Model 2: adjusted as in model 1 plus socioeconomic status (tertiles), education (high school, college, graduate), State (12 states of Mexico), menopausal status (premenopausal, postmenopausal, unknown), diabetes, hypercholesterolemia (yes/no), smoking (never, past, current, and missing), recreational physical activity (minutes/week), and energy intake (quartiles)

^d^Model 3: adjusted as in model 2 plus body mass index (kg/m^2^)

^e^Abbreviations. MTC: Mexican Teachers’ Cohort; Q1-Q4: quartile1-quartile4

To explore the consistency of the empirical dietary patterns we randomly divided the cohort into two groups and the dietary patterns derived in both groups resembled the three dietary patterns from the whole cohort. When we used an oblique rotation method and ran a maximum likelihood method it resulted in similar dietary patterns and similar associations with hypertension (data not shown). When we retained two components we obtained a similar Fruits & Vegetables pattern and Western pattern, also with similar associations with hypertension. However, using all individual food items on the food frequency questionnaire resulted in slightly different dietary patterns. The first component was similar (heavily loaded with fruits but also with nuts), the second component was different (loading heavily on vegetables), and the third component was similar to the Western pattern (loading heavily on processed meats and fast foods). The variance explained by these three components was 6.8%, 3.9%, and 3.3%, respectively. The third component was directly associated with hypertension while the first two were not (data not shown).

In sensitivity analyses we ran the multivariable adjusted model only in non-diabetics and non-smokers to reduce confounding (Additional file 1: Table S1). We also ran the multivariable adjusted model using the dietary patterns derived from the two component solution as the exposure. Among non-diabetics and using the two factor solution as the exposure results remained the same as in the main analyses. Among non-smokers results were slightly strengthened in the Fruits & Vegetables pattern as a protector factor and the Western pattern as high risk factor but were attenuated in the Modern Mexican pattern and results were no longer significant. When we used the less strict definition of hypertension, the associations with dietary patterns were stronger, in the same direction, and significant with all dietary patterns without changes in our main conclusions. Fruits & Vegetables pattern OR comparing extreme quartiles: 0.87 (95%CI: 0.79,0.96), *P*-trend: 0.002; Western pattern OR comparing extreme quartiles: 1.20 (95%CI: 1.08,1.34), *P*-trend: 0.0009; Modern Mexican pattern OR comparing extreme quartiles: 1.19 (95%CI: 1.07,1.32), *P*-trend: 0.001. Moreover, after further adjustment by BMI, as in the main analysis, the association with the Modern Mexican dietary pattern lost significance.

## Discussion

In this prospective study, the Western and Modern Mexican dietary patterns were directly associated with the incidence of hypertension in Mexican women. However, we did not observe an inverse association with the Fruits & Vegetables pattern (Prudent pattern) after adjusting for risk factors for hypertension.

The dietary patterns (Fruits & Vegetables and Western) we derived from PCA are consistent with Prudent and Western dietary patterns found in other studies [22–24] and in a previous MTC study [25]. However, besides these two globally similar patterns we also derived a third pattern which exemplifies the nutritional transition in Mexicans, who have low adherence to dietary recommendations [26]. Adherence to this pattern is characterized by eating traditional foods (i.e. corn tortillas and hot peppers) together with unhealthy foods (i.e. sodas) and low intake of fruits. The variance explained by the three dietary patterns (8.6%, 5.9% and 5.1%) is slightly lower than other dietary patterns derived by PCA in other studies [21, 22, 24]. However, when summing the variance explained by the three of them (19.6%), the total variance explained is within the range from other studies (17.4%–24%) [21, 22, 24].

It is difficult to compare empirically derived and a priori dietary patterns. Some of the advantages of using empirically derived dietary patterns are that they are data driven and allow for the examination of eating behaviors. Previous studies have shown inverse and direct associations between Prudent and Western patterns and cardiovascular risk, respectively [15, 21, 23]. Other studies have focused on the association between diet and blood pressure [12–14]. However, few have studied the relation between diet and incident hypertension and findings have been controversial [27–29]. While Schulze et al. did not find an association between exploratory dietary patterns and incident hypertension they did find an inverse association with the DASH diet (quartile 3 vs. 1) [27]. Li et al. found strong inverse associations between three commonly used index scores (Dietary Approaches to Stop Hypertension, alternative Mediterranean diet, alternative Healthy Eating Index) and hypertension but Toledo et al. found no association with fifteen hypothesis-oriented dietary patterns [28, 29]. However, all of them adjusted for BMI which we hypothesize is a mediator in the association between diet and hypertension. Adjusting for a mediator, in this case BMI, would cause collider bias [30] so the direct association between the dietary patterns and hypertension would not be valid.

Nutrients in fruits and vegetables hypothesized to lower blood pressure include fiber, potassium, magnesium, folate, vitamin C and flavonoids; however, other nutrient(s) or interactions among nutrients may be responsible for decreasing hypertension risk [31–35]. In our study we found no association between the Fruits & Vegetables pattern and hypertension; although we did find a suggestion of a protective effect only when the pattern score was modeled as a continuous variable. This was perhaps due to the fact that the beneficial effect of fruits and vegetables could have been obscured by grouping different types of fruits and vegetables, some of which may not have a beneficial effect on blood pressure [7, 8, 35]. Moreover, methods of cooking or added fats and seasonings could be counterbalancing the beneficial effects of vegetables. Additionally, the overall effect of fruits and vegetables on hypertension could be relatively small after considering other risk factors. The possibility of misclassification of the outcome and a short follow-up time may have also underestimated the association. Meat, processed meats, sweets and pastries have been associated with increased blood pressure [5, 35] perhaps due to their higher content of saturated fat, sodium, and sugar than fruits and vegetables [36–38]. Consistent with this literature, we found a direct association between both the Western pattern and the Modern Mexican pattern and incident hypertension. These findings are relevant because these patterns show frequently consumed foods (e.g. meat, processed meats, sweets and pastries) that can be targeted by public policy and decrease their intake.

### Strengths and limitations

Our study has several strengths, it is a prospective population-based study, with a large sample size, and multiple risk factors for hypertension were available to adjust for confounding, including smoking and other lifestyle factors. However, confounding by unmeasured or poorly measured factors cannot be ruled out. Self-reported diagnosis of hypertension has been used in other cohort studies and has been shown to be a valid indicator in Hispanics [39, 40]. The positive predictive value of self-report of treated hypertension was moderately high, thus measurement error is likely. This measurement error is probably non-differential since the exposure was ascertained before the outcome occurred. This error may have resulted in an underestimation of the association between dietary patterns and hypertension. A short follow-up time may have decreased the power to detect an association between the dietary patterns and hypertension, especially in the association with the Fruits & Vegetables pattern whose effect size may be smaller. However, this study has 80% power to detect an OR of 1.17 between Q4 and Q1 of each food pattern. The large sample size and population-based population were crucial in finding distinct dietary patterns which would be otherwise missed. However, dietary patterns derived by PCA depend on some arbitrary decisions that may affect the reproducibility of our findings such as the consolidation of food items into food groups, the number of components to retain, the rotation method, and the labeling of the components. We conducted sensitivity analyses to examine whether these decisions affected the consistency of our results by choosing alternatives and found similar dietary patterns and associations. We also split the sample into two groups and conducted the PCA in these random samples and found similar results. Using all food items changed the patterns derived. We hypothesize that when we used all food items in the PCA, results were different because we have many single vegetables (*n* = 20) and fruits (*n* = 20).

Since we did not have the certainty of the time of hypertension diagnosis we could not conduct a survival analysis, thus we had to exclude all participants which were lost to follow-up on the 2011 questionnaire and had to model the association with logistic regression as an alternative. Seventeen percent of the study population did not answer the 2011 follow-up questionnaire, which could cause selection bias. However, comparing response vs no response in the 2011 questionnaire (Additional file 1: Table S2) there were no major differences between these two groups, including the distribution of the dietary patterns scores. Since this study is restricted to Hispanic women, generalizability to other populations could be affected.

## Conclusions

In conclusion, we found evidence of a relation between the Western and Modern Mexican dietary patterns and hypertension in a population of Mexican women. These results are consistent with prior reports showing an association between a Western diet and coronary heart disease. However, our study also shows that a higher adherence to a nutritional transition diet, the Modern Mexican pattern, is also associated with hypertension. In contrast to previous findings, the Fruits & Vegetables pattern was not significantly associated with hypertension, probably due to misclassification of the outcome. Also, underestimation of the associations may have resulted from a short follow-up time. Our results should be confirmed in other Mexican based studies and evaluate longer term adherence to these dietary patterns. These finding have important public health implications, as improved dietary habits are crucial in the prevention of hypertension and other cardiovascular diseases among Mexicans living in Mexico, and possibly also Mexicans living in the USA.

## Additional file

Additional file 1: Table S1. Multivariate adjusted OR of incident hypertension by dietary pattern quartiles in women from the MTC. **Table S2.** Age-standardized characteristics of 75,006 Mexican women from the MTC at baseline by follow-up status. (DOCX 21 kb)
