# Supplementary material for: Western and Modern Mexican dietary patterns are directly associated with incident hypertension in Mexican women: a prospective follow-up study
Source: Nutr J. 2018 Feb 14;17:21. doi: 10.1186/s12937-018-0332-3 (PMC5813404; doi:10.1186/s12937-018-0332-3)
Supplement: Supplementary file 1 — Multivariate adjusted OR of incident hypertension by dietary pattern quartiles in women from the MTC. Table S2. Age-standardized characteristics of 75,006 Mexican women from the MTC at baseline by follow-up status. (DOCX 21 kb) [file 12937_2018_332_MOESM1_ESM.docx]

| Additional file 1: Table S1. Multivariate adjusted OR of incident hypertension by dietary pattern quartiles in women from the MTC^1-3^ | | | | | | |
| --- | --- | --- | --- | --- | --- | --- |
| Model | Q1 | Q2 | Q3 | Q4 | *P*-trend | Continuous^1^ |
|  | |  |  |  |  |  |
| *Non-smokers* | |  |  |  |  |  |
| Fruits & Vegetables | |  |  |  |  |  |
| Multivariable | 1 | 0.98(0.86,1.11) | 0.94(0.82,1.07) | 0.89(0.78,1.02) | 0.07 | 0.95(0.91,1.00) |
| Western |  |  |  |  |  |  |
| Multivariable | 1 | 1.06(0.94,1.21) | 1.11(0.97,1.26) | 1.29(1.12,1.48) | 0.0003 | 1.12(1.06,1.18) |
| Modern Mexican | |  |  |  |  |  |
| Multivariable | 1 | 1.08(0.95,1.23) | 1.20(1.06,1.37) | 1.11(0.97,1.28) | 0.06 | 1.03(0.98,1.08) |
|  |  |  |  |  |  |  |
| *Non-diabetics* | |  |  |  |  |  |
| Fruits & Vegetables | |  |  |  |  |  |
| Multivariable | 1 | 1.03(0.92,1.15) | 0.97(0.87,1.09) | 0.93(0.83,1.05) | 0.15 | 0.96(0.92,1.00) |
| Western |  |  |  |  |  |  |
| Multivariable | 1 | 1.11(0.99,1.24) | 1.13(1.00,1.27) | 1.24(1.10,1.40) | 0.0006 | 1.09(1.04,1.14) |
| Modern Mexican | |  |  |  |  |  |
| Multivariable | 1 | 1.12(1.00,1.25) | 1.19(1.06,1.33) | 1.15(1.02,1.29) | 0.01 | 1.05(1.00,1.09) |
|  |  |  |  |  |  |  |
| *Two factor solution* | |  |  |  |  |  |
| Fruits & Vegetables | |  |  |  |  |  |
| Multivariable | 1 | 1.01(0.91,1.13) | 0.93(0.84,1.04) | 0.96(0.86,1.07) | 0.28 | 0.96(0.93,1.01) |
| Western |  |  |  |  |  |  |
| Multivariable | 1 | 1.08(0.96,1.21) | 1.24(1.11,1.39) | 1.25(1.10,1.41) | ˂0.0001 | 1.10(1.05,1.14) |
|  |  |  |  |  |  |  |

^1^ Multivariate adjusted odds (95% CI) of incident hypertension by dietary patterns (continuous)

^2^ Multivariable model: adjusted for age (continuous), socioeconomic status (tertiles), education (high school, college, graduate), State (12 states of Mexico), menopausal status (premenopausal, postmenopausal, unknown), diabetes, hypercholesterolemia (yes/no), smoking (never, past, current, and missing), recreational physical activity (minutes/week), and energy intake (quartiles).

^3^ Abbreviations. MTC: Mexican Teachers’ Cohort; Q1-Q4: quartile1-quartile4

| Additional file 1: Table S2. Age-standardized characteristics of 75,006 Mexican women from the MTC at baseline by follow-up status^1-4^ | | |  |
| --- | --- | --- | --- |
|  | Answered follow-up questionnaire 2011 | |  |
|  | Yes  (*n*=62,913) | No  (*n*=12,093) | P-value |
| Age at questionnaire^2^, years | 42.1±7.3 | 42.7±8.2 | ˂0.0001 |
| Indigenous^3^, % | 8 | 10 | ˂0.0001 |
| Regions in Mexico |  |  | ˂0.0001 |
| - Northern Mexico, % | 20 | 19 |  |
| - Central Mexico, % | 16 | 14 |  |
| - Mexico City and State of Mexico, % | 26 | 19 |  |
| - Southern Mexico, % | 38 | 48 |  |
| Graduate education, % | 14 | 11 | ˂0.0001 |
| Socioeconomic status, tertiles |  |  | ˂0.0001 |
| - Tertile 1, % | 25 | 32 |  |
| - Tertile 2, % | 32 | 31 |  |
| - Tertile 3, % | 44 | 38 |  |
| Menopausal status |  |  | ˂0.0001 |
| - Premenopausal, % | 76 | 74 |  |
| - Postmenopausal, % | 15 | 16 |  |
| - Missing, % | 9 | 10 |  |
| Body mass index, kg/m² | 27.0±4.4 | 27.0±4.4 | 0.86 |
| Current smoker, % | 9 | 10 | ˂0.0001 |
| Diabetes mellitus, % | 3 | 3 | 0.052 |
| Hypercholesterolemia, % | 11 | 10 | 0.70 |
| Recreational physical activity, min/week | 60(0,210) | 60(0,210) | 0.53 |
| Total energy, kcal/day | 1736(1358,2201) | 1721(1332,2215) | 0.06 |
| Protein, % energy | 16.3±2.8 | 16.3±2.7 | 0.76 |
| Fat, % energy | 28.7±5.5 | 28.8±5.4 | 0.004 |
| Carbohydrate, % energy | 57.3±7.9 | 57.1±7.7 | 0.01 |
| Fruits & Vegetables Dietary Pattern, score | -0.025(0.986) | -0.016(0.992) | 0.69 |
| - Quartile 1, % | 25 | 25 | 0.051 |
| - Quartile 4, % | 25 | 25 |  |
| Western Dietary Pattern, score | -0.022(1.048) | 0.009(0.991) | 0.0002 |
| - Quartile 1, % | 25 | 27 | ˂0.0001 |
| - Quartile 4, % | 25 | 25 |  |
| Modern Mexican Dietary Pattern, score | 0.009(1.019) | -0.002(0.993) | 0.77 |
| - Quartile 1, % | 25 | 25 | 0.09 |
| - Quartile 4, % | 25 | 26 |  |

^1^Values are mean ± SD for continuous variables and percent for categorical variables. Physical activity and total energy intake are expressed as median (IQR). Values are age standardized to the age distribution of the study population. Values of polytomous variables may not sum to 100% due to rounding.

P-values are reported testing for the equality of means or proportions using t-test or chi-square as appropriate.

^2^ Variable is not age adjusted

^3^ Participants who reported speaking an indigenous language or having a parent who did were defined as indigenous

^4^ Abbreviations. MTC: Mexican Teachers’ Cohort;
